# Safety and effectiveness of bariatric surgery: Roux-en-Y gastric bypass is superior to gastric banding in the management of morbidly obese patients

**DOI:** 10.1186/1754-9493-3-10

**Published:** 2009-05-29

**Authors:** Ulrich Guller, Lazar V Klein, John A Hagen

**Affiliations:** 1 Center for Excellence in Bariatric Surgery, Humber River Regional Hospital Finch Site, University of Toronto, Department of Surgery, Toronto, ON, M3N 1N1, Canada; 2 Department of Surgery, Division of Visceral Surgery and Transplantation, Inselspital, University of Bern, 3010 Bern, Switzerland

## Abstract

**Background:**

The use of bariatric surgery in the management of morbid obesity is rapidly increasing. The two most frequently performed procedures are laparoscopic Roux-en-Y bypass and laparoscopic gastric banding. The objective of this short overview is to provide a critical appraisal of the most relevant scientific evidence comparing laparoscopic gastric banding versus laparoscopic Roux-en-Y bypass in the treatment of morbidly obese patients.

**Results and discussion:**

There is mounting and convincing evidence that laparoscopic gastric banding is suboptimal at best in the management of morbid obesity. Although short-term morbidity is low and hospital length of stay is short, the rates of long-term complications and band removals are high, and failure to lose weight after laparoscopic gastric banding is prevalent.

**Conclusion:**

The placement of a gastric band appears to be a disservice to many morbidly obese patients and therefore, in the current culture of evidence based medicine, the prevalent use of laparoscopic gastric banding can no longer be justified. Based on the current scientific literature, the laparoscopic gastric bypass should be considered the treatment of choice in the management of morbidly obese patients.

## Background

The prevalence of obesity as well as its associated morbidity and mortality are rising at an alarming rate in industrialized countries [1-3]. This has a major public health impact as morbid obesity is associated with diabetes, arterial hypertension, hypercholesterolemia, sleep apnea syndrome, arthritis, and decreased life expectancy. Unfortunately, attempts to lose weight with dieting, behavioural modifications, and exercise are unsuccessful in the vast majority of morbidly obese patients. Therefore, different bariatric surgery procedures have been developed. The number of performed bariatric procedures have rapidly and considerably increased over the past decade [4-6]. Interestingly, a landmark study recently published in the New England Journal of Medicine demonstrated that bariatric surgery results in a decreased overall mortality in morbidly obese patients[7].

Although there are a variety of different bariatric surgery procedures, the most frequently performed and best studied are laparoscopic gastric banding and laparoscopic Roux-en-Y gastric bypass [4-6]. These two procedures differ significantly in many ways: the gastric band is exclusively a restrictive procedure, whereas the gastric bypass has both restrictive and malabsorptive features. Furthermore, the laparoscopic implantation of a gastric band is technically much less challenging, does not require any intra-corporeal anastomosis, and is often performed by general surgeons without specific training for laparoscopic and/or bariatric surgery. Conversely, the laparoscopic gastric bypass is a high-end laparoscopic procedure as it entails 2 intra-corporeal anastomoses (an entero-enterostomy and a gastro-jejunostomy), requires the mastering of intra-corporeal suturing skills, and is usually performed by surgeons with advanced laparoscopic or specifically laparoscopic-bariatric surgery training.

The use of laparoscopic gastric banding may seem appealing at first as the rate of early post-operative complications is low and the hospital stay short[8]. However, there have been numerous reports on long-term complications such as band slippage and migration, pouch and esophageal dilation, port-site infection, and failure to lose weight, all of which frequently require the band removal [8-16].

The objective of this short overview is to provide a critical appraisal of the most important scientific evidence comparing laparoscopic gastric banding versus laparoscopic Roux-en-Y bypass for patients with morbid obesity.

## Methods

The online databases Pubmed, Cochrane library, and Google Scholar were searched to identify all relevant literature regarding laparoscopic banding and Roux-en-Y gastric bypass surgery for morbid obesity published up to March 31st, 2009. The key terms gastric banding, laparoscopy, gastric bypass, Roux-en-Y gastric bypass, LAGB, RYGBP, bariatric surgery, bariatric procedure were used in combination and independently for the literature search. No limits to language, type of article, country of origin, gender, and publication date were imposed. References from systematic reviews were manually searched for articles not identified using the search engines. Furthermore, several experts in the field were contacted to identify important unpublished data.

## Results and discussion

Despite the prevalent use of bariatric surgery during the past years, there is only one randomized controlled trial comparing laparoscopic banding versus laparoscopic Roux-en-Y bypass. In this small trial from an Italian group[17], 51 patients were randomly assigned to laparoscopic adjustable gastric band (n = 27) versus Roux-en-Y gastric bypass (n = 24). The mean operating time was significantly shorter and there were less short-term complications in the banding group. However, after a five year follow-up, patients undergoing Roux-en-Y bypass surgery had significantly lower BMI and an increased percentage of excess weight loss compared to the banding patients. Conversely, weight loss failure (defined as a BMI > 35 kg/m2) was significantly more prevalent in the gastric band group than in bypass patients (34.6% versus 4.2%, p < 0.001). This difference is not only statistically significant but also of tremendous clinical relevance for both patients and health care providers.

Tice et al. recently published a well-researched and well-performed systematic review comparing bypass and band[8]. Fourteen studies (including the previously mentioned randomized controlled trial) with a follow-up of at least one year comparing Roux-en-Y gastric bypass versus laparoscopic adjustable gastric band were summarized. While operating room time and length of hospital stay were shorter in the banding group, loss of excess body weight was consistently and statistically significantly better in patients undergoing laparoscopic Roux-en-Y bypass (median difference: 26%, range 19% to 34%, p < 0.001). Furthermore, the rate of resolution of comorbid diseases such as diabetes, arterial hypertension, dyslipidemia, sleep apnea syndrome, and osteoarthritis clearly favoured laparoscopic Roux-en-Y bypass. The peri-operative mortality was low (less than 0.5%) for both procedures. Most importantly, re-operation rates were clearly lower and patient satisfaction significantly higher in the bypass group. Based on these results, the authors concluded that laparoscopic gastric bypass should be the primary bariatric procedure in the management of morbid obesity.

The vast majority of scientific reports on laparoscopic gastric banding have a short follow-up, which limits their usefulness and scientific value as long-term weight loss and long-term complication and re-operation rates are of utmost importance. However, there have been several recent publications on laparoscopic gastric bands that report a long-term follow up. Suter and colleagues summarized their prospectively collected results on 317 patients undergoing laparoscopic banding[13]. Long-term follow up was excellent (88.2% at 5 years). One third (33.1%) of their patients developed late complications such as band erosion, pouch dilation, band slippage, and catheter and port related problems. Major re-operations were required in 21.7% of all patients and the failure rate consistently increased from 23.8% at 3 years to 31.5% at 5 years, up to 36.9% at 7 years. The 7-year success rate (defined as excessive weight loss of more than 50%) was extremely low (43%). Based on these concerning and disappointing results, the authors concluded that "laparoscopic banding should no longer be considered as the procedure of choice for obesity"[13].

Some studies have reported acceptable intermediate-term results for patients undergoing laparoscopic gastric banding [18-20]. Two systematic reviews by an Australian group demonstrate that laparoscopic gastric banding is associated with considerable weight loss in the medium term[19,20]. In deed, O'Brien and colleagues nicely summarized all reports on bariatric procedures with a follow up equal to or greater than 3 years and with an initial sample size of at least 100 patients[19]. They report that laparoscopic Roux-en-Y bypass was associated with significantly greater weight loss than laparoscopic gastric banding during the first two years of follow up but no statistically significant difference was found at 3 and more years of follow-up. However, this review has some major limitations as acknowledged by the authors, including an unknown percentage of patients who were lost to follow up as well as a dramatic decrease of sample sizes after four years of follow up. Moreover, none of these reviews report the long-term complication and long-term re-operation rates. In fact, the literature on long-term outcomes of patients undergoing laparoscopic gastric banding is scarce but several investigations report a considerable increase in complications and band removal with increasing follow-up[12,13,16].

There are numerous other investigations that report rates of gastric band removal up to 60%[8,9,11-16,21]. The rapidly increasing body of scientific evidence on high rates of re-operation after laparoscopic gastric banding is alarming. An increasing number of reports describe the conversion from gastric banding to other bariatric procedures including laparoscopic gastric Roux-en-Y bypass, sleeve resection, and duodenal switch[22,23].

Proponents of the laparoscopic gastric band argue that improvements in the surgical technique (e.g. pars flaccida technique) and the quality and design of bands have considerably reduced the number of complications. While this is true for band slippage [24-26], the rate of long-term complications, including band removals, remain high[12,13,16].

Different studies have attempted to define criteria of morbidly obese patients who are unlikely to benefit from a laparoscopic gastric banding. Woelnerhanssen et al. reported their single-institutional experience of 380 morbidly obese patients. A multivariable analysis was performed, which revealed that patients with binge eating disorders, sweat-eating behaviour, as well as elderly patients may be poor candidates for laparoscopic gastric banding[27]. In a recently published case series of 448 patients with an average follow-up of over 3 years[12], a BMI over 50 kg/m2 was associated with a greater risk for reoperation. Patients with lower BMI and those who are able to change their eating habits are most likely to benefit from a laparoscopic gastric banding[28].

## Conclusion

In summary, there is rapidly mounting and convincing evidence that laparoscopic gastric banding is suboptimal in the management of morbid obesity. Although short-term complications are low and hospital length of stay is short, the long-term problems including band removals are high and failure to lose weight after laparoscopic gastric banding is prevalent. The placement of a gastric band appears to be a disservice to many morbidly obese patients and therefore, in the present day and age of evidence based medicine, the frequent use of laparoscopic gastric banding can no longer be justified. Based on the current scientific literature, the laparoscopic gastric bypass should be considered the treatment of choice in the management of morbidly obese patients.

## Competing interests

The authors declare that they have no competing interests.

## Authors' contributions

JH and LK conceived the study and participated in its design. UG drafted the manuscript. All authors read and approved the final manuscript.
